# Supplementary material for: Labor market segmentation and the gender wage gap: Evidence from China
Source: PLoS One. 2024 Mar 28;19(3):e0299355. doi: 10.1371/journal.pone.0299355 (PMC10977760; doi:10.1371/journal.pone.0299355)
Supplement: S1 Text — (DOCX) [file pone.0299355.s001.docx]

**Supporting Information**

**S1 Detailed introduction to Collective Economy**

In China, the collective economy represents a form of economic organization and a mode of ownership. It exists mainly in rural areas. The following are some aspects of what the collective economy represents in China.

Rural collective economy. Rural areas consist mainly of farmers who conduct agricultural production and business through collective economic organizations. Rural collective economy means that farmers are organized under collective ownership and work together to manage resources such as farmland, forest land, pastureland, and fisheries, as well as operating and managing them professional cooperatives and cooperative economic organizations. The collective economy plays an important role in rural areas, contributing to the modernization of agriculture, the growth of farmers' incomes and the development of the rural economy.

Autonomy and development. Collective economy is also closely related to villagers autonomy and shared development in China. Villagers usually participate in decision-making and management of affairs through institutions such as village committees. Collective economies provide common benefits to villagers and improve village infrastructure, public services, and social welfare through the distribution and use of collective economy revenues.

Rural Land System. Collective ownership is one of the basic institutional forms of China land system. According to the Chinese Constitution, land is owned by the state, collectives, or individuals. The rural collective economy plays an important role in land use and management, enabling farmers to conduct agricultural production on collective land through, for example, land contract management rights and specialized cooperatives.

Rights and interests protection. The collective economy also provides a mechanism for farmers to protect their rights and interests. Through collective economic organizations, farmers are able to unite for land rights, economic benefits, and social security. The collective economy plays an active role in promoting the protection of farmers' rights and interests, social stability, and rural social governance.
