# Supplementary material for: Labor market segmentation and the gender wage gap: Evidence from China
Source: PLoS One. 2024 Mar 28;19(3):e0299355. doi: 10.1371/journal.pone.0299355 (PMC10977760; doi:10.1371/journal.pone.0299355)
Supplement: S1 Table — (DOCX) [file pone.0299355.s002.docx]

**Supporting Information**

**S2 Industry-Classification in 2004, 2008 and 2013**

**Table A Industry-Classification in 2004, 2008 and 2013**

| 15-category Industry (2004) | 20-Category Industry (2008/2013) |
| --- | --- |
| Agriculture | Agriculture, forestry, animal husbandry and fishery |
| Mining | Mining |
| Manufacturing | Manufacturing |
| Electricity, Gas and Water | Electricity, gas and water |
| Construction | Construction |
| Water and Environment Management | Transportation, warehousing, and postal industry |
| Transport and Information | Information transmission; computer service and software |
| Wholesale and Retail, Hotel and Restaurants | Wholesale and retail |
| Financial Intermediation | Accommodation and Catering |
| Real Estate | Financial |
| Households and Business Services | Real estate |
| Health, sports and social welfare | Leasing and business services |
| Education, culture and broadcast | Scientific research, technical services, and geological prospecting industry |
| Scientific Research | Water conservancy, environment, and public facilities management |
| State and social organization | Resident services and other services; Education; Health, Social welfare; Culture, sports, and entertainment; Public Administration and Social  Organization |

*Source:* China Statistical Yearbook by province (2004, 2008 and 2013), China Labor Statistical Yearbook (2004, 2008 and 2013)
