# Supplementary material for: Labor market segmentation and the gender wage gap: Evidence from China
Source: PLoS One. 2024 Mar 28;19(3):e0299355. doi: 10.1371/journal.pone.0299355 (PMC10977760; doi:10.1371/journal.pone.0299355)
Supplement: Attachment — Submitted filename: rebuttal letter .docx [file pone.0299355.s004.docx]

**Dear Editor and Reviewers:**

Thank you for your letter and for the reviewers’ comments concerning my manuscript entitled “Labor market segmentation and gender inequality: evidence from China”. This revision of the manuscript took us over a year. Partly due to the sudden opening of China's lock down last year, but more importantly, we have carefully and strictly followed each of your comments and have rewritten almost the entire article. From abstract to conclusion, the workload is enormous.

Those comments are extremely valuable and very helpful for revising and improving our paper, as well as the important guiding significance to my research. The main responses and to comments of editor and each reviewer are as flows.

Because we rewrote the article, therefore we do not submit the Tracked Manuscript, and the line are totally according to the new submitted one.

**To Editor**

Q1: After my careful reading of the paper, I agree with the second reviewer that your analysis is potentially interesting and the results may be a new contribution to the gender inequality literature in China, but the current version of the paper suffers from a number of limitations and as stated by the first reviewer, it is not effective in documenting properly the existence of gender inequalities in the labor market. Although I believe it is a risky revision, I decided to give you the opportunity to thoroughly revise the paper following all the detailed comments of both the Reviewers.

A1: Thank you for your valuable comment. First, thank you very much for giving us this opportunity to make revisions. On the one hand, we have rewritten the full text and try to solve limitations in accordance with the reviewer's comments, and on the other hand, we have utilized a new model (BFG model, which takes into account the fact that workers can choose among the public sector, the private sector, and the collective economy instead of two) to draw different conclusions from the previous ones, considering of heterogeneity of the population with different wage groups. Therefore, our paper can make a substantial contribution to literature on gender inequality in China.

Q2: I agree with both reviewers that the review of the literature and the descriptive statistics included the paper are off-center and should be focused on gender wage differentials, and that also the introduction should refer to studies on gender inequality in the labor market in China and in other countries as well.

A2: Thank you for your valuable comment. First, we rewrote the introduction section to open with a direct reference to gender inequality. Second, we rewrote the literature review section to include much more literature on gender inequality and the sector. Third, we remade the descriptive statistical tables to wage groups by sector and gender to fit the focus of our study.

Q3: Furthermore, when using the Heckman Selection model identification through the functional form is no more acceptable and some exclusion restrictions should be included in the selection equation.

A3: Thank you for your valuable comment. We abandoned the use of the Heckman sample selection model and instead recalculated the results using the Bourguignon, F., Fournier, M. and Gurgand (BFG) model and modified our conclusions and policy recommendations based on the results.

Q4：Secondly, as suggested by Reviewer 1, I believe that most of your empirical exercises are not convincing and should be improved. In particular, I agree that the selection into a specific sector should not be treated as independent, as it is in the paper, but a more comprehensive approach should be adopted. There exist many possible empirical solutions, as reviewed by Bourguignon et al (2007) [ Bourguignon, F., Fournier, M. and Gurgand, M. (2007), SELECTION BIAS CORRECTIONS BASED ON THE MULTINOMIAL LOGIT MODEL: MONTE CARLO COMPARISONS. Journal of Economic Surveys, 21: 174-205. https://doi.org/10.1111/j.1467-6419.2007.00503.x].  Among these, you can choose the most suitable to you setting and data.

A4：Thank you for your valuable comment. We read the article carefully and realized that traditional techniques to address the issue of self-selectivity, such as the Heckman sample selection model and the treatment effects model, are inherently limited to bivariate cases. Then we used BFG (Bourguignon, F., Fournier, M. and Gurgand, M) model as you guided. This BFG model accommodates a polychotomous selection process, thereby allowing for multiple categories. Further, we adjusted our conclusions and policy recommendations based on the findings of the BFG, which makes our article one of the few papers that utilize the BFG to study the gender wage gaps in China.

Q5：Lastly, but most importantly, I do agree with Reviewer 1 that the English of the paper is very poor, and you should seek independent editorial help before submitting a revision.

A5：Thank you for your valuable comment. We used a proofreading service after we finished writing the entire article, and the entire text has been checked by a native English speaker to help us with grammar and other issues, which makes our research article much smoother to read.

Q6: Please let me remark that, given the two referee reports, the revision process will actually require to deeply revise your empirical strategy and to rewrite substantially your manuscript.

A6: Thank you for your valuable comment. After carefully reading the comments of two reviewers, we rewrote the full text including tables, figures, models, and regressions. Our article has been greatly and substantially improved both in terms of breadth and depth.

**To Reviewers**

**Reviewer 1**

Q1: The article analyzes gender inequality in China distinguishing between three potential employers: public sector, private sector, and collective agreements. They found that while gender differences are prevalent in all sectors, the size and time patterns differ. While the topic is relevant, and potentially interesting, the execution requires further refinement before the paper is ready for publication.

Overall, authors perform several econometric estimations, but each of them lacks depth. The authors do not scratch beyond the surface, and as a result the analysis is simplistic. Authors could, among others, explain how wages are determined in the public and collective sector. Are they based on market-like mechanisms or are wages based on other considerations?

A1：Thank you for your valuable comment.

First, we carefully followed your opinions and revised each piece of comment in detail. In short, we rewrote the background to sector and gender, introduced the mechanism, added a lot of literature, reworked the tables and figures, regressed using the BFG model and discussed inter- and intra-sectoral differences using Brown wage decomposition. In other words, we nearly rewrote a new article.

Second, for mechanisms, we first discussed the role of collective in the introduction section (line 74 - line 94), then we carefully stated the sector and wage determination in. China (line 189 – line 242), which discuss the difference between market-like mechanisms and administration-like mechanisms influencing the wages from perspective of gender. This discussion gives us foundation of later results discussion and (line 697 – line 648) and policy suggestions (line 761-line 798).

**Major concerns**

Q2: The authors should consider using professional proofreading service before submitting the paper. As it stands, it contains sentences that are difficult to read, or plainly awkward. This wording complicates evaluating the merits of the paper, as it oftentimes it is not clear what authors are referring to.

A2: Thank you for your valuable comment. We used a proofreading service after we finished writing the entire article, and the entire text has been checked by a native English speaker to help us with grammar and other issues, which makes our research article much smoother to read.

Q3: Where is gender? For a paper that attempts to be an analysis of gender inequality, gender is notoriously absent. The authors do not reference / relate to recent literature on gender inequality. Throughout the paper, one can find bits where it appears that what authors really want to study is differences across sectors, and that the inclusion of gender is an afterthought. I think this is a serious flaw in their analysis. While the debates around roots of gender inequality might be different in developing countries and rural areas (when compared to Europe and North America), one should bring these to the table. The coefficients by themselves do not tell a story.

A3: Thank you for your valuable comment. We totally rewrote the whole article with totally focusing on the gender inequality. First, in the introduction section, we directly mentioned the situation of female in China and how literature ignores the gender wage inequality by sector (line 35 – line 142). Second, we talked about the sector segmentation and wage determination with focusing on how this will be causing gender wage gaps (line 190 – line 242). Third, in literature review section, we dropped articles not relevant enough to gender study, and concentrate on gender wage gaps in China with comparing developed country and developing countries (line 243 – line 391). Furthermore, we made more tables and figures to clarify the situation of gender wage gaps by sector. After regression, we discussed results (line 696 – line 747) and also came up with matched policy suggestions to gender wage gaps under Chinese context (line 760 – line 797).

Q4: The dependent variable is annual income. The variable is problematic if men and women work different hours to cope with household responsibilities (e.g., in Germany mothers are more likely to work part-time than fathers), or if women experience more work interruptions throughout the year, for example if they work in more seasonal positions, or in positions with more churning. Working with hourly wages, provided that they are available, would be better. If not, authors should at least discuss how working with annual data affects the estimates of the gender wage gap. Notice, that my concerns are related to the functioning of labor markets in developed countries and might not apply to China.

A4: Thank you for your valuable comment.

First, we are extremely sorry that we do not have related data about wage composition. We have two sources, the in Urban Household Survey (UHS) conducted by the National Bureau of Statistics of China, and the China Trade Union Statistical Yearbook, compiled by the All-China Federation of Trade Unions. We use them because both the UHS and the China Trade Union Statistical Yearbook are nationally recognized source though the latter ceased updates after 2013 (that is also why we choose 2004, 2008 and 2013). We further discuss it in line 395 – line 401.

Second, in order to further clear up the relation between sector, wage and gender, we remade the tables (Table 2 – Table 3) to help readers understand how annual data affects the estimates of the gender wage gap. Furthermore, we made Figure 1 – Figure 2 focusing on wage distribution and wage quantiles by a clear comparison of genders in three sectors of year 2004, 2008 and 2013.

Third, in 6.2 limitation section, we admit that we do not have much more information about wages such as monthly wages and subsidies (line 798 – line 813). This deficiency becomes critical given the varying welfare benefits across sectors in China, resulting in an incomplete picture of the gender wage gap.

Q5: The selection models.

The authors implemented a Heckman correction model to study the returns to characteristics in different sectors. However, the model is based on the selection into employment, where workers become employees if market wages are above reservation wages. The problem that they study differs from the simple selection model in that the alternative to work in private sector is not being out of employment. The alternative is work in a different sector. Using a logic from Roy's model, they work in the private sector if their expected income (or utility, depending on how the model is characterized) is higher than what they would receive in any of the remaining two sector. Following this reasoning, the estimation should account for the three alternatives in the estimation.

The problem then is carried to the empirical estimation. The authors estimate the three outcomes as if they were independent, whereas a joint estimation would better suit the data and the proposed analysis. By estimating regressions separately by sector, authors throw away valuable information: they discard the data on wages actually perceived by workers. In other words, having information on wages in public sector serves not only to estimate the returns to characteristics in that sector, but also in private and collective sectors. Authors can refer to Heckman and Honore (1990) for references on the estimation of Roy models.
Another issue with these models concerns identification. When using sample selection models, the parameters are identified either through exclusion restrictions or through functional form assumptions. The latter option was chosen by the authors. This choice is problematic, as it is much more demanding that the use of exclusion restrictions. It requires that the functional form is indeed correct for consistency. From the earlier discussion, I believe that this is not the case. My recommendation on this issue is to consider including some exclusion restrictions, such as education of the parents.

A5: Thank you for your valuable comment. We used the new model (BFG model), made new regressions and discussed new results and came up with new policy recommendations supervised by you. With your guidance, after reading Heckman and Honore (1990) and Bourguignon, F., Fournier, M. and Gurgand, M. (2007), we realized that traditional techniques to address the issue of self-selectivity, such as the Heckman sample selection model and the treatment effects model, are inherently limited to bivariate cases. In our study the choices include the public sector, private sector, and collective economy. Therefore, we dropped all the content about Heckman selection model. Instead, we choose to use BFG model as this model accommodates a polychotomous selection process based on the multinomial logit model, thereby allowing for multiple categories. This part includes methods (line 507 – line 540)，the regression discussion (line 600 – line 635) and Table 4. We are really sorry that we do not have data such as education of the parents.

Q6: The Oaxaca-Blinder model.

This section really suffers from the lack of clarity. Using jargon that is common to the literature (unexplained instead of unexplainable) would benefit the paper. Besides this comment, authors need to address additional issues.

1) Why does the selection disappear? The previous subsection was about selection into working in each sector. However, when moving to the OB decomposition concerns over selection are muted.

2) The interpretation of the coefficients is not clear. For example, authors write that "collective economy had the smallest characteristic effect among the three sectors." The value was actually negative. Following a standard decomposition, this value is given by b(X_m - X_f), and the correct interpretation would be that had the workers been rewarded based on the same schedule, then women should receive higher wages.

3) Comparing estimates of the gender wage gap over time and across sectors is difficult if personal characteristics are not constant. Otherwise changes in estimates reflect both changes in the estimated parameters (beta) and the mean characteristics in the sector period (X's). If selection is relevant (and based on characteristics), then the estimates of gender wage gap might be different across sectors, even if betas are identical.

A6: Thank you for your valuable comment.

First, after careful consideration, we abandoned the use of OB decomposition and used Brown decomposition. The OB decomposition usually considers two sectors, while not considering inter- and intra-sectoral differences, but the Brown decomposition takes sector segregation into account in the wage decomposition, i.e., it decomposes gender earnings differentials into intra- and inter-occupational differentials. In line 568 – line 597, we carefully stated the content of Brown decomposition.

Second, we double check the terminology about the wage decomposition. For example, in line 568 – line 597, we carefully discussed the four parts of Brown decomposition focusing on “explained”, “unexplained”, “differential”, etc., .

Third, after using brown wage decomposition, we carefully discussed about the results (line 664 – line 694). We discussed the results from four parts and gave the matched policy recommendations in section 6.1 (line 761 – line 797).

Q7: The RIF regression
The authors do not discuss the results; they enumerate the coefficients from the table. A proper discussion of the differences in wage inequality over distributions by sector would be welcomed.

A7: Thank you for your valuable comment.

First, we expressed the results in section 5.2 (line 637 – line 663) with double check the grammar and using more precise terminologies. The RIF regression results are shown in Table 5.1 – Table 5.3. The results statements are mentioned in line 638 – line 650. Second, we discussed the results shortly in line 652 – line 662. Finally, with the results of RIF regressions, I further talked about the results in section 5.4, especially in the first two paragraphs (line 696 – line 731).


**Minor comments**

Q8: The authors are not consistent in defining terms in the model description. In the Heckman model, they define D* to be a latent variable, but they later use P_j which depends on P*. Then, when defining expected wages they introduced additional parameters that were not mentioned earlier (sigma and rho). In the Oaxaca-Blinder model, the authors decompose the gap between workers in the public and the private sector, which does not match the actual decompositions that they run.

A8: Thank you for your valuable comment.

Now our empirical methods are mainly consisted of four parts, and for each we checked carefully about the expression and symbols we use to help reader understand the methods easier. We have not only noticed about the contents, but also corrected the formats of variables and formulars.

In section 4.2.1, we introduced the Mincer equation (line 492 – line 505). In section 4.2.2, we introduced BFG model with equation 4.21 – equation 4.2.6 (line 507 – line 540). Then in section 4.2.3, we talked about the recentered influence function (RIF) regression (line 541 – line567). And finally, we stated the detailed content on Brown decomposition (line 568 – line597).

Q9: Code. While the author's willingness to share the code is commendable. By looking at the codes, we learn about problems with the estimation that would have gone mute. Particularly, both the Oaxaca-Blinder regression and the Recentered Influence Functions treat occupation and industries as continuous variables, when they are clearly categorical. For this reason, I abstracted from commenting the results from their research. The shared codes also fail to allow an independent replication. The data has arguably been preprocessed, it has only the variables used in the analysis and only observations from actually working individuals. While this is sufficient for reproducing the results from the table, it is not sufficient to ensure transparency of the research process. I would like authors to include, among others, references to the raw database (DOI if available), information on how to access these data and codes for data curation.

A8: Thank you for your valuable comment.

We really appreciate for your opinions about our codes and data, but we are very sorry that Urban Household Survey (UHS) is conducted by the National Bureau of Statistics of China and now is stored by Tsinghua China Date Center（CDC）at Tsinghua University. Now the UHS stops to update in 2015 and the China Trade Union Statistical Yearbook, which is a nationally recognized source, ceased updates after 2013.

The data can be requested from Tsinghua China Date Center（CDC）and we do not have enough authority.

<http://www.tcdc.sem.tsinghua.edu.cn/en/index.htm>

Therefore, we have no authority to provide all the data and can only submit the data after collection. Furthermore, we remade Table 2 – Table 3, Figure 1 – Figure 2 that show the basic statistical description and wage distribution by sector and quantile.

As for codes, now we use occupation and industries are calculated as category variables into all the regressions in BFG, RIF regressions and Brown decomposition. As it is the first article using BFG model to research on gender wage gaps in China, we could potentially need some more time to publish the codes.

**Reviewer 2**

Q1：This study focused on the development of gender wage inequalities across different sectors. The authors applied Heckman’s two-stage method and presented the wage equations by sectors to solve the selection bias of working in different sectors. Further, this study used the Oaxaca-Binder decomposition method to analyze sector wage differences. By adding the average gender wage gap as one explanatory variable, the authors concluded the differences and development of the gender wage gap across sectors. Finally, this study used quantile regression to investigate the influence of each industry on quantile wages.

Overall, this paper clearly explains its questions, hypothesis, and research methods. However, its inferences and discussion present nonnegligible flaws, which might make its contributions doubtable. A few aspects/questions regarding the methods, inferences, and writings follow.

**Overall assessment:**

Q1：This study focused on the development of gender wage inequalities across different sectors. The authors applied Heckman’s two-stage method and presented the wage equations by sectors to solve the selection bias of working in different sectors. Further, this study used the Oaxaca-Binder decomposition method to analyze sector wage differences. By adding the average gender wage gap as one explanatory variable, the authors concluded the differences and development of the gender wage gap across sectors. Finally, this study used quantile regression to investigate the influence of each industry on quantile wages.

Overall, this paper clearly explains its questions, hypothesis, and research methods. However, its inferences and discussion present nonnegligible flaws, which might make its contributions doubtable. A few aspects/questions regarding the methods, inferences, and writings follow.

A1: Thank you for your valuable comment. It is your supervision and encourage that support us to finish such large workload (almost rewrote the whole article). We carefully revised the entire article according to your comments piece by piece.

In short, we firstly rewrote the introduction part focusing on gender wage gaps context in China and point out the deficiencies in current literature (line 34 – line 88) with our substantial and schematic contribution and findings. Second, in order to help the reader to better understand the history and Chinese context, we introduced the mechanism and wage determination mechanism by sector in section 2 (line 189 – line 242). Third, we added a lot of literature with dropping irrelevant citations concentrate on China wage gaps (line 243 – line 390). Fourth, we introduced new model (BFG instead of Heckman; Brown instead of OB wage decomposition) with reworking all the tables and figures (line 395 – line 597). After analyzing the new regressed results, by BFG model, RIF model and Brown decomposition method (line 600 – line 747). Lastly, after discussion the results we give the conclusion by giving matched policy recommendations (line 748 – line797) and limitations (line 798 – line 813). In addition, we totally redesign the figures and tables.

In other words, we nearly rewrote a new article with giving substantial contribution from both width and breadth.

Because we rewrote the article, therefore we do not submit the Tracked Manuscript, and **the lines and sections are totally according to the new submitted one.**

**Main comments/questions:**

Q1: In the wage equation, age and/or its squared term are encouraged to be added.

A1: Thank you for your valuable comment. As we have already the “working experiences” as our control variable, it will cause collinearity problem if we add extra “age” as control variables (as age is highly related to working experience). Therefore, we do not put age as our control variables, but we do use “squared term of working experiences” in traditional Mincer income equation in the regression and we find the most coefficients are significant.

Q2: This article meant to explore the wage gaps across sectors and by genders; however, the descriptive statistics did not show those detailed wage gaps; authors can make a table to summarize the (gender) wage gaps by different sectors with significance test.

A2: Thank you for your valuable comment.

First, we totally remake the Table 2 and Table 3 In Table 2, we give the Descriptive Statistics by Gender; in Table 3, we give a careful description of Gender Wage by Sector. At the same time, in Table 3, I use T-test to confirm that the differences between the male and female are truly different as P-value is mostly less than 0.01.

Second, we remake the figure 1 and figure 2. In figure 1 (a), (b), (c), we draw the Kernel density distribution of gender wages by sector in year 2004, 2008 and 2013. In figure 2, we draw the wage gaps and gender wage ratio by percentile in 2004, 2008 and 2013. The figures clearly show the absolute and ratio of wages between the women and men by sector.

Third, in line 411 – line 490, we state Table 2 – Table 3, Figure 1 – Figure 2 clearly in words, which can help the readers to understand quickly and easily.

Q3: For line-300 to line-302, two questions: 1) are the income level adjusted in two years due to inflation? 2) the logarithmic form of wage is vital to compare as it indicates the relative changing ratio instead of the absolute values.

A3: Thank you for your valuable comment. First, the income levels of two years are adjusted based on year 2004. Second, in Figure 1 and Figure 2 (especially Figure 2), we draw the figures focusing on gender wage gaps by sector in different years and ratio by quantile in different years by sector. Third, we both discuss the wage gaps changes in line 411 – line 490.

Q4: In section 4.1: Result of Heckman selection model, the discussion of the selection bias of joining different sectors is problematic: 1) why do you believe there was a selection bias regarding incomes by joining different sectors; 2) can you provide valid references/data to persuade readers there was a selection bias among those sectors in the said years and said provinces; 3) normally at least one exogenous identification variable should be added into the selection function; 4) it is more persuaded if authors could expound their evidence that how the variables “marriage status” and “education” had become the identification variables. 5) the negative coefficient of “education” tends to be caused by insufficient selection correction. 6) the results without Heckman selection correction can also be presented as a contrast so the analysis will be more informative and receivable.

A4: Thank you for your valuable comment.

Frist, after second consideration, we decide to use the new model (BFG model), made new regressions, and discussed new results and came up with new policy recommendations supervised by you. After reading Heckman and Honore (1990) and Bourguignon, F., Fournier, M. and Gurgand, M. (2007), we realized that traditional techniques to address the issue of self-selectivity, such as the Heckman sample selection model and the treatment effects model, are inherently limited to bivariate cases.

In our study the choices include the public sector, private sector, and collective economy. Therefore, we dropped all the content about Heckman selection model. Instead, we choose to use BFG model as this model accommodates a polychotomous selection process based on the multinomial logit model, thereby allowing for multiple categories.

As guided by you, we show that “marriage status” and “education” had become the identification variables and education in most situations are playing positive roles. This detailed content about BFG model includes methods (line 507 – line 540)，the regression discussion (line 600 – line 635) and the detailed results are shown in Table 4 with are variables needed. The matched results discussion is in line 697 – line747, which we also give matched policy suggestions in the last section.

Q5: In section 4.2: Result of Blinder–Oaxaca decomposition. 1) Authors are encouraged to try different reference coefficients and reference groups in the decomposition; specifically, the option of “pooled/omega” of the “Oaxaca” syntax in STATA can also be considered; 2) as no obvious hint, was the Heckman selection model considered into the BO decomposition? 3) if authors wanted to compare the gender wage gaps among sectors, then decomposing sector wage differentials may not be a good idea. The gender wage gap could be an explanatory variable to induce the gaps between sectors; however, the latter is limited to reversely explaining the former without valid causal inferences: the explanation in line-371 is untenable.

A5: Thank you for your valuable comment. After careful consideration, we abandoned the use of OB decomposition as there are many disadvantages and used Brown decomposition. The OB decomposition usually considers two sectors (which matched the Heckman selection model), while it does not fit under three sectors context nor not considering inter- and intra-sectoral differences, but the Brown decomposition takes sector segregation into account in the wage decomposition, i.e., it decomposes gender earnings differentials into intra- and inter-occupational differentials. In line 568 – line 597, we carefully stated the content of Brown decomposition. we double check the terminology about the wage decomposition. For example, in line 568 – line 597, we carefully discussed the four parts of Brown decomposition focusing on “explained”, “unexplained”, “differential”, etc. After using brown wage decomposition, we carefully discussed about the results (line 664 – line 694). We discussed the results from four parts and gave the matched policy recommendations in section 6.1 (line 761 – line 797).

Q6: In section 4.3, the comparison of coefficients needs to be more cautious.

A6: Thank you for your valuable comment. First, we expressed the results now in section 5.2 (line 637 – line 663) with double check the grammar and using more precise terminologies. The RIF regression results now are shown in Table 5.1 – Table 5.3. The results statements are mentioned in line 638 – line 650. Second, we discussed the results shortly in line 652 – line 662. Finally, with the results of RIF regressions, I further talked about the results in section 5.4, especially in the first two paragraphs (line 696 – line 731). We also checked the other grammar and terminologies of other paragraphs.

**Writing tips：**

Q7: The introduction about gender wage inequalities lacks evidence and references, especially in paragraphs one and two.

A7: Thank you for your valuable comment. We supplement and revise the gender wage inequalities from different aspects in this article. First, in the first paragraph, we directly point out that the gender wage gaps are enlarging during last two decades (line 35 – line 47). Then we came up with idea that why is it urgent to make research on this topic (line 53 – line 142). Third, we introduce the gender wage gaps background and history factor in section 2 (line 189 – line 242). In literature review part, we cite many articles to show the existence of gender wage gaps (line 338 – line 390). Furthermore, the figures and tables are supportive to show that gender wage inequalities in. China.

Q8: Table 1. Classification of Sectors and Types of Occupations on page-2 is seemingly redundant. Most subjects can be combined with Table 2 on page-6, and some can be expressed through table notes.

A8: Thank you for your valuable comment. First, we totally drop the Table 1 as we have discussed the concepts of the three sectors in the first introduction section and section wage determination sector more or less. Second, we improved remade the Table 2 (now it is Table 1) and put the industries as Appendix at the end of the tables.

Q9: In line-214, the headline for RIF regression is incorrect.

A9: Thank you for your valuable comment. We use the proofreading service and checked by ourselves to ensure that there are no grammar and headline order mistakes in the whole article to make the research smoother to read.

Q10: It would be more informative if a Male-Female or cross sectors t-test could be considered in Table 3 on page-7, which better conveys the main idea of the present article. Table 4 and Table 5 could be combined as one table

A10: Thank you for your valuable comment. In Table 3, we not only supplement the wage description by three sectors in year 2004, 2008 and 2013, but also we calculate the pair-t test to test if there are significant wage differences between the male and the female among the sectors.

We guess you mean “Table 3 and Table 4” can be combined; therefore we combine the two tables and now it is Table 2. The table 2 are consist of two pages, which includes most relevant the variables. As guided by you, we also express some explanation on variables through table notes.
